# Toxicity Assessment of Metyltetraprole, a Novel Fungicide Inhibitor, to Embryo/Larval Zebrafish (*Danio rerio*)

**DOI:** 10.3390/toxics13080634

**Published:** 2025-07-28

**Authors:** Taylor Casine, Amany Sultan, Emma Ivantsova, Cole D. English, Lev Avidan, Christopher J. Martyniuk

**Affiliations:** 1 Center for Environmental and Human Toxicology, Department of Physiological Sciences, College of Veterinary Medicine, University of Florida, Gainesville, FL 32611, USA; tcasine@ufl.edu (T.C.); amanysultan2025@gmail.com (A.S.); eivantsova@ufl.edu (E.I.); coleenglish@ufl.edu (C.D.E.); lavidan@ufl.edu (L.A.); 2 Animal Health Research Institute, Agriculture Research Center (ARC), Giza 3725004, Egypt; 3 UF Genetics Institute, Interdisciplinary Program in Biomedical Sciences Neuroscience, University of Florida, Gainesville, FL 32611, USA

**Keywords:** fungicides, strobilurins, behavior, gene expression, zebrafish

## Abstract

Strobilurins are a prominent class of fungicides capable of entering aquatic environments via runoff and leaching from the soil. Findings from previous studies suggest that strobilurins are highly toxic in aquatic environments, and evidence of acute developmental toxicity and altered behavioral responses have been emphasized. The objective here was to determine the effects of a new strobilurin, metyltetraprole (MTP), on zebrafish using developmental endpoints, gene expression, and behavioral locomotor assays. We hypothesized that MTP would cause developmental toxicity and induce hyperactivity in zebrafish (*Danio rerio*). To test this, developing zebrafish embryos/larvae were exposed to environmentally relevant levels of MTP (0.1, 1, 10, and 100 µg/L) until 7 days post-fertilization. Survival percentages did not differ among the treatment groups. No change in reactive oxygen species production was detected, but two genes involved in the mitochondrial electron transport chain (*mt-nd3* and *uqcrc2*) were altered in abundance following MTP exposure. Moreover, the highest concentration (100 µg/L) of MTP caused notable hyperactivity in the zebrafish in the visual motor response test. Overall, results from this study increase our knowledge regarding sub-lethal effects of MTP, helping inform risk assessment for aquatic environments.

## 1. Introduction

Used globally as agrochemicals, strobilurins are a popular fungicide class that accounts for over 20% of the fungicide market [1]. Due to their high effectiveness against a variety of fungal diseases, strobilurins are commonly applied to a broad spectrum of crops to manage fungal pathogens and, due to their relatively high water solubility, strobilurin residues enter aquatic environments via runoff or leaching from soil following agricultural applications [2]. In fact, azoxystrobin has been detected globally at concentrations up to 34 μg/L in various bodies of water [3]. Another prominent strobilurin, pyraclostrobin, has been reported at 0.73 μg/L in Australia [4] and 1.61 μg/L in Nebraska, United States [5]. The ability of strobilurins to contaminate water sources raises concerns over the potential risk to aquatic organisms, especially when considering that an estimated 2.5 million pounds (1130 tons) of azoxystrobin and pyraclostrobin were used in the United States in 2018 according to the United States Geological Survey [6].

Referred to as quinone outside inhibitors (QoIs) due to their mechanism of action, strobilurins prevent the transfer of electrons at the ubiquinol oxidizing (Qo) site [7]. Specifically, strobilurins bind the Qo site of cytochrome b in complex III of the mitochondrial respiratory chain to suppress mitochondrial respiration and inhibit the production of ATP [8]. Despite being deemed relatively non-toxic to birds, mammals, and humans, strobilurins display high toxicity to aquatic organisms [7]. Prior studies have focused on the lethal and sub-lethal effects of strobilurins on aquatic species. Results indicate that strobilurins hinder mitochondrial bioenergetics in zebrafish, resulting in oxidative stress and apoptosis [7]. Various studies also report that strobilurins can alter the behavior of zebrafish, causing hyperactivity at low doses and hypoactivity at high doses [2,9], and can contribute to cardiotoxicity, genotoxicity, neurotoxicity, immunotoxicity, and endocrine disruption [10].

While many studies have been conducted to determine the lethal and sub-lethal effects of a variety of strobilurins, the impact of metyltetraprole (MTP) on non-target aquatic organisms requires further examination. Developed in response to increasing QoI-resistant plant pathogenic fungi, MTP is a newly synthesized strobilurin considered to be the first of a new generation of strobilurins, which is composed of a 3-substituted central ring containing tetrazolinone moiety [11]. This structure allows MTP to avoid steric hindrance between the strobilurin and mutant target site, enabling it to combat QoI-resistant mutants. Studies suggest that despite containing a similar side chain to a known strobilurin and pyraclostrobin, MTP’s unique tetrazolinone structure allows it to be highly effective against strains that are resistant to pyraclostrobin and other strobilurin fungicides [12]. Regarded as a promising new fungicide, MTP may become critical in managing diseases affecting crops [12]; however, studies have not yet been conducted to determine the potential biological impacts of MTP exposure to aquatic organisms.

This study aimed to assess the toxic and sub-lethal effects of environmentally relevant doses of MTP on zebrafish (*Danio rerio*) embryos/larvae. Zebrafish are commonly used as toxicity models and are effective in determining the lethal and sub-lethal effects of pesticides [13]. Relevant environmental concentrations used in this study were estimated based on a closely related strobilurin, pyraclostrobin. Developmental endpoints, such as survivorship, hatch rate, and deformities, were measured. In addition, behavioral responses were recorded to determine if MTP affects zebrafish locomotion. Based on data for other strobilurins [2,9], we hypothesized that MTP would result in acute developmental toxicity and induce hyperactivity in zebrafish. Results from this study will inform risk assessment of novel strobilurin fungicides like MTP in aquatic ecosystems.

## 2. Materials and Methods

### 2.1. Chemical Preparation

Metyltetraprole (CAS 1472649-01-6, purity > 95%) was purchased from Millipore-Sigma (St. Louis, MO, USA). Stock solutions of MTP were prepared in 0.1% dimethyl sulfoxide (DMSO, CAS 67–68-5, Millipore-Sigma). Embryo rearing medium (ERM) was used to dissolve MTP [final nominal concentrations of 0.1, 1, 10, or 100 μg/L MTP] with a final concentration <0.1 % *v/v* DMSO. Recipes for ERM can be found in Westerfield [14].

### 2.2. Maintenance and Egg Production of Zebrafish

Adult zebrafish (AB × Tübingen, *Danio rerio*) from the University of Florida were used as outlined previously [15,16]. Rearing and staging of the zebrafish embryo followed Kimmel et al. [17]. Additional details on housing and breeding are presented in Supplementary Materials. The Institutional Animal Care and Use Committee of University of Florida approved all experiments (UF IACUC#201708562).

### 2.3. Metyltetraprole Exposure Regime

Fertilized and normally developing embryos were selected at ∼6 h post-fertilization (hpf) using a dissecting microscope. Zebrafish embryos were assigned in random fashion into experimental groups (ERM, 0.1% DMSO, or one dose of 0.1, 1, 10, or 100 μg/L MTP). Six experiments were conducted using embryos that were generated from separate breeders of fish (glass petri dishes or glass beakers in each experiment housed 20–40 embryos in 20 mL of ERM with or without chemicals). The biological replicate was petri dish or beaker depending on which vessel was used in the exposure. Storage of chemicals and deformity scoring is detailed in Supplementary Materials.

### 2.4. Reactive Oxygen Species

Embryos were obtained for ROS assessment as outlined above in Section 2.2. ROS was conducted on 7-day old fish. Complete methods are presented in Supplementary Materials and followed the method of [18]. The exposure concentrations were 0.1% DMSO, 1 µg/L, 10 µg/L, or 100 µg/L MTP (*n* = 4 petri dishes per experimental group). ROS levels were normalized to total protein (µg/mL) as determined by a Bradford assay.

### 2.5. Real-Time PCR

Zebrafish embryos at 6 hpf were exposed to 0.1% DMSO, ERM, or one dose of 0.1, 1, or 10 μg/L MTP for gene expression analysis. Each beaker contained 20 embryos and exposure conditions were maintained as that above. Following the 7-day exposure period, larvae were pooled within a beaker, anesthetized (buffered MS-222), and subjected to liquid nitrogen to be housed at −80 °C prior to RNA extraction. Supplementary Materials have details on real-time PCR assays, and the method has been previously published by us [19]. Primers used in this study were obtained from the published literature (Supplemental Table S1) [20,21,22,23,24]. Three housekeeping genes (ribosomal subunit 15, *rps15*, 18s ribosomal rRNA, *18s*, and beta actin, *b*-actin) were used for normalizing expression. The qPCR analysis included four “no reverse transcriptase” samples and two “no template control” samples. Sample sizes ranged between 3 and 7 across treatments for gene expression analysis.

### 2.6. Locomotor Activity

Three independent experiments were performed to test the dark photokinesis response in 7-day old larvae. Data from each trial were normalized to a mean control value of 1 (relative movement to control), and then data were combined into a single graph. In each trial, zebrafish embryos at 6 hpf were randomly assigned to an experimental group 0.1% DMSO, ERM, or one dose of 0.1, 1, or 10 μg/L MTP (*n* = 20–24 individuals/treatment). The assay proceeded as per our previous methods [25] and details are given in Supplementary Materials.

### 2.7. Statistical Analysis

Statistical analysis and graphing were conducted with GraphPad PRISM V10 (La Jolla, CA, USA). A Mantel–Cox test was employed to analyze survival. For ROS (log transformed), gene expression analysis (log10 transformed data), and locomotor activity, a one-way ANOVA was employed followed by a Dunnett’s post hoc test. For behavior analysis, each time period (3 dark and 2 light periods) was analyzed as a discrete response. The DMSO solvent group was considered the control for comparison. There was no difference between the negative control (ERM only) and DMSO control for any endpoint. For all endpoints, the significance of difference was considered at *p* < 0.05.

## 3. Results

### 3.1. Survival and Deformity

**Figure 1:**
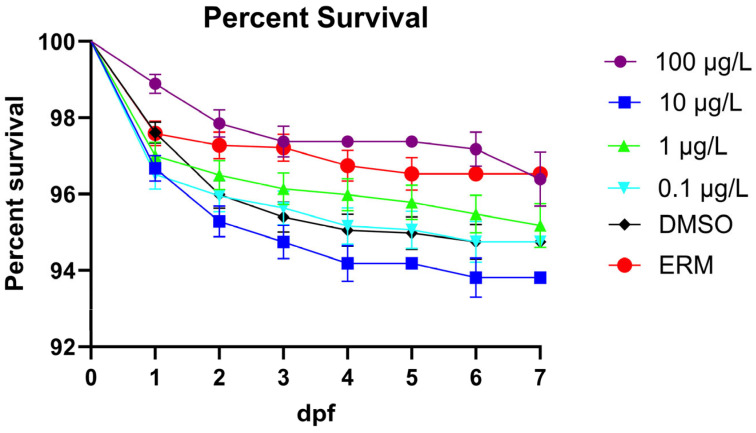
Proportion of surviving zebrafish embryos/larvae following a 7-day exposure to ERM, 0.1% DMSO, 0.1, 1, 10, or 100 µg/L metyltetraprole.

Six separate experiments were used to gather data for developmental endpoints. The combined survival rates showed no significant difference between the DMSO control and the concentrations of MTP (Log-rank (Mantel–Cox test), Chi-square = 30.98, d.f. = 5, *p* ≤ 0.0001). Instances of mortality were relatively low across all treatment groups (Figure 1). Few deformities were recorded in each treatment group (>5%). There was a small decrease in survival with 10 µg/L relative to the ERM but overall, survival was high (94–97% for all groups) and not significantly impacted by metyltetraprole up to 100 µg/L. While there were no significant reoccurring deformities (less than 3%), spinal curvature, axial malformations, yolk sac edema, and pericardial edema were noted but these occurred across different treatments with no relationship to concentration.

### 3.2. Reactive Oxygen Species

**Figure 2:**
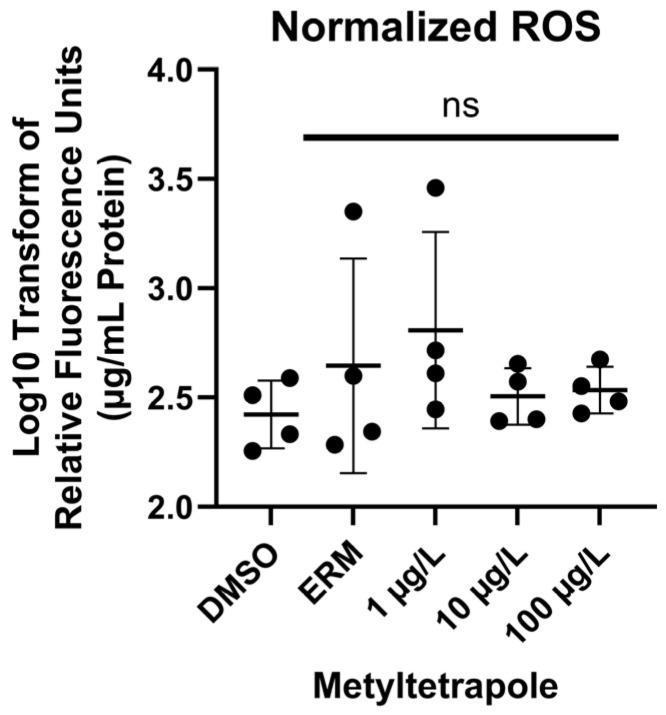
Reactive oxygen species in 7 dpf zebrafish exposed to 0.1% DMSO, ERM, 1, 10, or 100 µg/L metyltetraprole (MTP) expressed as log10-transformed relative fluorescence units (per µg/mL protein). The mean value of the group (±SD) is indicated by the horizonal line and each circle is a biological replicate (one-way ANOVA, Dunnett’s multiple comparisons test, *n* = 4 biological replicates per treatment). ns = not significant.

There was no significant effect of MTP on ROS levels [F_(4, 15)_ = 0.9010, *p* = 0.4879] (Figure 2) at any concentration tested.

### 3.3. Mitochondrial- and Oxidative Stress-Related Transcripts

**Figure 3:**
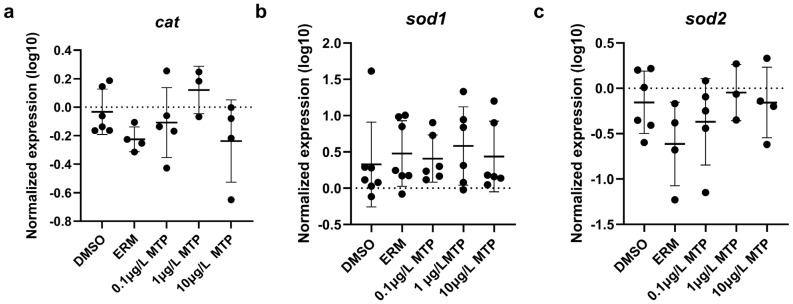
The expression levels of (**a**) *cat*, (**b**) *sod1*, (**c**) *sod2*, in 7-day old larval zebrafish exposed to 0.1% DMSO, ERM, 0.1, 1, or 10 µg/L metyltetraprole (MTP). Each point is a biological replicate, and the horizontal line indicates the mean value of the group (mean ± S.D.) (one-way ANOVA with a Dunnett’s multiple comparisons test, ERM (*n* = 4–7), 0.1% DMSO (*n* = 6–7), 0.1 µg/L MTP (*n* = 5–6), 1 µg/L MTP (*n* = 3–6), 10 µg/L MTP (*n* = 4–6)).

**Figure 4:**
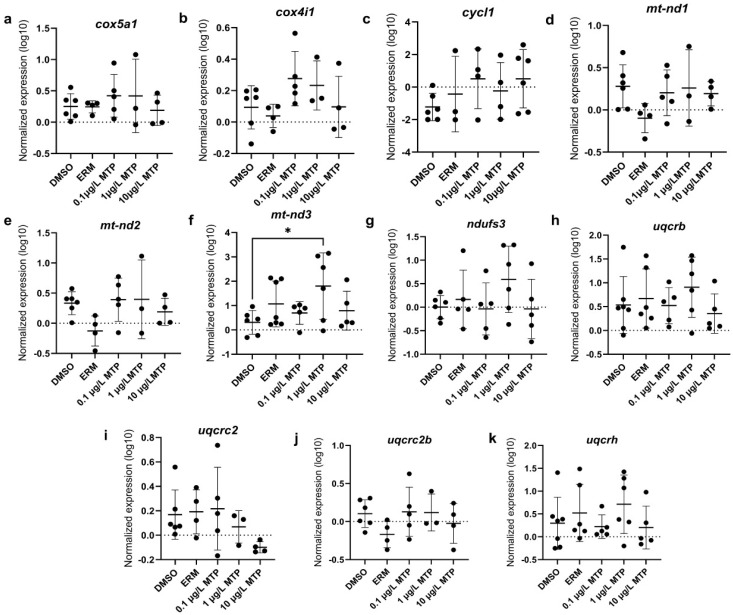
The expression levels of (**a**) *cox5a1*, (**b**) *cox4i1*, (**c**) *cycl1*, (**d**) *mt-nd1*, (**e**) *mt-nd2*, (**f**) *mt-nd3*, (**g**) *ndufs3*, (**h**) *uqcrb*, (**i**) *uqcrc2*, (**j**) *uqcrc2b*, and (**k**) *uqcrh* in 7-day old larval zebrafish exposed to either ERM, 0.1% DMSO, 0.1, 1, or 10 µg/L metyltetraprole (MTP). Each point is a biological replicate, and the horizontal line indicates the mean value of the group (mean ± S.D.) (one-way ANOVA with a Dunnett’s multiple comparisons test, 0.1% DMSO (*n* = 6–7), ERM (*n* = 3–7), 0.1 µg/L MTP (*n* = 4–5), 1 µg/L MTP (*n* = 3–6), 10 µg/L MTP (*n* = 4–6)). Asterisk denotes significant difference at * *p* < 0.05 from solvent control.

**Figure 5:**
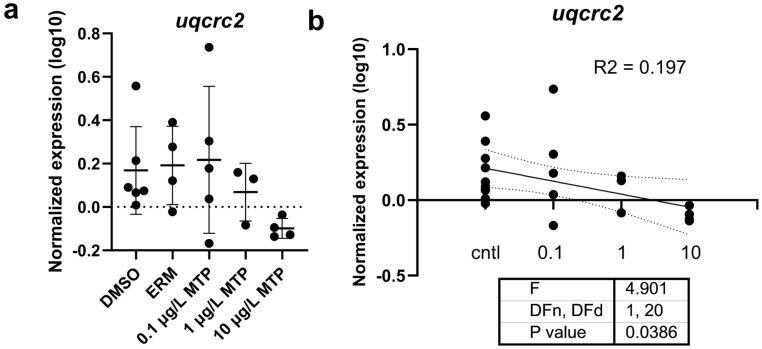
(**a**) The expression level of *uqcrc2* in 7-day old larval zebrafish exposed to 0.1% DMSO, ERM, 0.1, 1, or 10 µg/L metyltetraprole (MTP). Each point is a biological replicate, and the horizontal line indicates the mean value of the group (mean ± S.D.) (one-way ANOVA with a Dunnett’s multiple comparisons test, DMSO (*n* = 6), ERM (*n* = 4), 0.1% 0.1 µg/L MTP (*n* = 5), 1 µg/L MTP (*n* = 3), 10 µg/L MTP (*n* = 4)). (**b**) Regression analysis demonstrating a significant reduction in *uqcrc2* expression with increasing concentration of MTP.

Regarding oxidative stress-related transcript levels, *cat*, *sod1*, and *sod2* were not different among groups (Figure 3). Mitochondrial-related transcript levels of *cox5a1*, *cox4i1*, *cycl1*, *mt-nd1*, *mt-nd2*, *ndufs3*, *uqcrb*, *uqcrc2*, *uqcrc2b*, and *uqcrh* were not different among groups; however, *mt-nd3* was significantly upregulated in fish exposed to 1 µg/L MTP (*p* < 0.05) (Figure 4). The expression level of *uqcrc2* in 7-day old larval zebrafish exposed to 0.1% DMSO, ERM, 0.1, 1, or 10 µg/L metyltetraprole (MTP) also changed in a dose-dependent manner (Figure 5). A regression analysis revealed a reduction in *uqcrc2* expression with increasing concentration of MTP.

### 3.4. Behavioral Assessment

**Figure 6:**
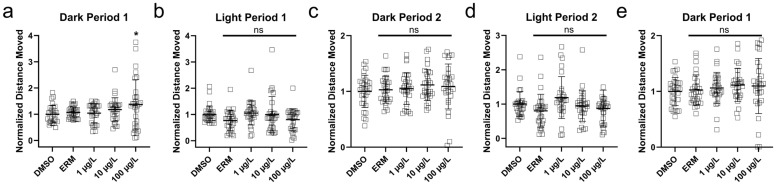
Distance moved (log-transformed) of zebrafish larvae 7 days post-fertilization that were exposed to 0.1% DMSO, ERM, 1 μg/L, 10 μg/L, and 100 μg/L metyltetraprole (MTP). Each point depicts one fish. (**a**) First dark period, (**b**) first light period, (**c**) second dark period, (**d**) second light period, (**e**) third dark period. Dunnett’s multiple comparison tests were used to determine differences among groups. Significant differences were noted at * *p* < 0.05. ns = not significant.

Three independent trials were conducted for VMR, and data for distance travelled was combined for each experiment (Figure 6). Hyperactivity was observed in larval zebrafish following 100 µg/L MTP during the first dark period (F_(4,145)_ = 7.960, *p* < 0.0001). Treatment groups during dark periods 2 and 3 and all light periods did not present with changes in locomotive activity relative to the DMSO control.

## 4. Discussion

Several studies have investigated the effects of different strobilurins, reporting a range of sub-lethal and lethal effects, including developmental delays, mitochondrial dysfunction, behavioral abnormalities, and altered gene expression [2,7,9,10]. As MTP is a relatively new strobilurin, there is a lack of data on its adverse effects in non-target species. To our knowledge, this is the first study to report on the impact of MTP on fish. Thus, research on the effects of MTP on aquatic organisms is warranted. To evaluate sub-lethal and lethal effects due to exposure, developmental endpoints (i.e., survival, hatch frequency, deformities) were measured. A behavioral assay was conducted to elucidate the effects of MTP on locomotor activity. As no toxicity studies of MTP are available on other aquatic organisms or rodents, we have gathered toxicity data on other strobilurin fungicides, including azoxystrobin and fenamidone.

When evaluating the survivorship of zebrafish embryos/larvae across three experiments, no significant difference in mortality relative to the control was observed for 0.1 μg/L, 1 μg/L, 10 μg/L, and 100 μg/L MTP. This is consistent with other studies investigating the toxicity of strobilurins, which show that the mortality rates are generally less notable at relatively low concentrations. One study reports that the concentration of azoxystrobin required for significant mortality in developing zebrafish may exceed 1000 μg/L [26] and Kumar et al. [27] also reports that 1000 mg/L azoxystrobin did not induce mortality in zebrafish embryos; however, 1000 mg/L pyraclostrobin caused 100% mortality. In regard to pyraclostrobin, severe mortality was observed in concentrations as low as 81.3 μg/L [28], and concentrations of 58.6 μg/L were reported to delay hatch rates [29]. When exposed up to 48 µg/L pyraclostrobin from 4 to 8 dpf, mortality was not induced in zebrafish larvae exposed to less than 33 µg/L and mortality reached 88% at 48 µg/L [30]. In our study with MTP, results showed no significant delays in hatching at all tested doses, suggesting a higher dose may be needed to impact hatch rates. Across the three experiments, deformities were relatively infrequent (>5%) and no significant reoccurring malformations were detected. This is consistent with the literature, which reports that azoxystrobin concentrations of 500 μg/L are needed to induce significant deformities in zebrafish embryos/larvae [31]. Huang, Souders II [9] noted pericardial edema, yolk sac edema, and spinal curvature in zebrafish exposed to at least 2.5 µM fenamidone for 5 days. Kumar et al. [27] also reported no deformities in zebrafish exposed to 0.1–100 mg/L azoxystrobin or pyraclostrobin from 4 hpf to 48 hpf. Other studies, however, report that pyraclostrobin can cause significant deformities in zebrafish at concentrations as low as 25 μg/L [31] and all zebrafish treated with 73.0 μg/L pyraclostrobin had malformations [29]. Additionally, Li et al. [30] found 55% of surviving zebrafish larvae treated with 44 µg/L pyraclostrobin from 4 to 8 dpf exhibited brain damage and pericardial edema. Overall, no overt toxicity was detected based on survivorship, hatch rate, and deformities for 0.1 μg/L, 1.0 μg/, 10 μg/L, or 100 μg/L MTP.

At the concentrations tested, we did not detect any significant changes in ROS levels nor antioxidant transcripts among groups, but we did detect changes in expression levels of *mt-nd3* and *uqcrc2*. Both genes play essential roles in the mitochondrial electron transport chain, contributing to efficient cellular energy production. Specifically, *mt-nd3* is involved in Complex I by contributing to the initial step of oxidative phosphorylation, while *uqcrc2* is involved in Complex III by transferring electrons from ubiquinol to cytochrome c. Other studies report reduced mitochondrial activity following exposure to strobilurins. Huang, Souders II [9] exposed zebrafish to 0.1–5 µM fenamidone for 5 days. Oxygen consumption rates were evaluated and both basal and ATP-linked respiration were decreased in fish exposed to 2.5 µM and 5 µM fenamidone. Additionally, maximal respiration was decreased in fish exposed to 5 µM and non-mitochondrial respiration was decreased in fish treated with 1, 2.5, and 5 µM fenamidone. Various genes related to oxidative stress and apoptosis were measured following zebrafish exposure to 100 nM to 2 µM fenamidone and only sod2 was altered in which levels were significantly reduced at the highest tested concentration. Qin et al. [32] exposed zebrafish to 5, 50, 200, or 500 ng/L fenbuconazole for 96 h. Mitochondrial membrane potential and Complex II and III activities were reduced by 200 and 500 ng/L. Additionally, 500 ng/L fenbuconazole reduced basal OCR and oligomycin-induced ATP. ROS were increased at 50 ng/L and higher and total antioxidant capacity was decreased at all tested concentrations in a concentration-dependent manner. MDA levels increased at 500 ng/L fenbuconazole. Kumar et al. [27] exposed zebrafish embryos to 0.1, 10, 100 mg/L azoxystrobin or pyraclostrobin from 4 hpf to 48 hpf. Mitochondrial function was disrupted by 100 mg/L pyraclostrobin in which basal and maximal respiration at 48 hpf were significantly reduced. mRNA transcripts related to oxidative stress or apoptosis were not altered. Additionally, malondialdehyde (MDA) levels and caspase 3/7 activity were unremarkable following azoxystrobin and pyraclostrobin exposure. When exposed up to 36 g/L pyraclostrobin until 8 dpf, zebrafish had suppressed mitochondrial complex III and IV activities with 36 μg/L pyraclostrobin and complex V activity was inhibited with 18 and 36 μg/L [30]. Additionally, ATP content was significantly reduced by exposure to 18 and 36 μg/L.

Behavioral analysis often yields valuable insight into the effects of chemicals on locomotor activity and neuronal function. Zebrafish have a natural response to light and dark cycles that reflect predator–prey responses and disruptions in this response can be detrimental for fish. Our VMR data revealed that 100 μg/L MTP induces hyperactivity in zebrafish larvae during the first initial dark phase. Conversely, the locomotor activity was not significantly different in light phases. These data are consistent with other strobilurins. Huang, Souders II [9] and Li, Qin [2] report that hyperactivity occurred in dark periods for each of the following strobilurins: azoxystrobin, kresoxim-methyl, trifloxystrobin, and pyraclostrobin. Zhu et al. [33] found that exposure to doses as low as 0.1 μg/L caused hyperactivity in a species of minnow, *Gobiocypris rarus*. Contrasting the hyperactivity that is observed at low concentrations, high concentrations of strobilurins have been shown to cause hypoactivity [27,30]. Specifically, 36 g/L pyraclostrobin reduced the average velocity and distance moved [30] and reduced total distance traveled and average velocity with 1000 mg/L azoxystrobin and 100 pyraclostrobin mg/L in zebrafish larvae [27]. Previous findings suggest that variations in activity levels are closely linked to neurotoxicity [34,35]. Liang et al. [35] suggest that alterations to neurotransmitter signaling in zebrafish caused anxiety-like behaviors, such as hyperactivity, when exposed to the synthetic phenolic antioxidant butylated hydroxytoluene. In these fish, dopamine signaling was notably inhibited, causing increased locomotion. Alternatively, disruptions in mitochondrial bioenergetics may be related to the hyperactivity that is observed at low concentrations [24]. Specifically, studies suggest that altered expression levels of genes located in the mitochondrial complex may be the cause of hyperactivity and hypoactivity in zebrafish exposed to strobilurins [9]. Further studies are needed to assess the root cause of the observed hyperactivity in MTP. It appears zebrafish larvae initially respond to the dark period with hyperactivity at the highest concentration tested, but this effect is dissipated over time.

## 5. Conclusions

In conclusion, this study investigated the sub-lethal and lethal effects of MTP in developing zebrafish. In general, there were no significant differences in survivorship, hatch rate, or deformities for the concentrations studied. Results from the VMR study revealed significant increases in hyperactivity. Additional studies focused on assessing neurotransmitter systems and mitochondrial bioenergetics for MTP are needed to evaluate the etiology of the observed hyperactivity. Overall, the results of this study will help guide future research to better understand the risk assessment of MTP in aquatic environments.

## Data Availability

All data is provided in the manuscript.

## Supplementary Materials

The following supporting information can be downloaded at https://www.mdpi.com/article/10.3390/toxics13080634/s1, Table S1: Primers used for real-time PCR analysis.
