# Supplementary material for: Toxicity Assessment of Metyltetraprole, a Novel Fungicide Inhibitor, to Embryo/Larval Zebrafish (Danio rerio)
Source: Toxics. 2025 Jul 28;13(8):634. doi: 10.3390/toxics13080634 (PMC12390205; doi:10.3390/toxics13080634)
Supplement: Supplementary file 1 [file toxics-13-00634-s001.zip › Suppl. Methods.pdf]

toxics

MDPI

Article

Toxicity assessment of metyltetraprole, a novel fungicide inhibitor, to embryo/larval zebrafish (*Danio rerio*)

Taylor Casine [1], Amany Sultan[1,2], Emma Ivantsova[1], Cole D. English [1], Lev Avidan [1], Christopher J. Martyniuk[1,3*]

[1] Center for Environmental and Human Toxicology, Department of Physiological Sciences, College of Veterinary Medicine, University of Florida, Gainesville, Florida, 32611, USA
[2] Animal Health Research Institute, Agriculture Research Center (ARC), Giza 3725004, Egypt
[3] UF Genetics Institute, Interdisciplinary Program in Biomedical Sciences Neuroscience, University of Florida, Gainesville, FL 32611, USA
* Correspondence: cmartyn@ufl.edu

2. Materials and Methods

2.1 Chemical Preparation

Metyltetraprole (CAS 1472649-01-6, purity > 95%) was purchased from Millipore-Sigma. Stock solutions of MTP were prepared in 0.1% dimethyl sulfoxide (DMSO, CAS 67–68-5, Millipore-Sigma). Exposure solutions were prepared in embryo rearing medium (ERM) to yield final nominal concentrations of 0.1, 1, 10, or 100 μg/L MTP with a final concentration < 0.1 % v/v DMSO. Recipes for ERM can be found in Westerfield [1].

2.2 Maintenance and egg production of zebrafish

Adult zebrafish (AB x Tübingen, *Danio rerio*) were raised in a flow-through Pentair system in the Cancer-Genetics Research Center at the University of Florida as outlined previously [2, 3]. The University of Florida maintains a breeding colony for research and outbreeds with extraneous fish to maintain high genetic diversity. Rearing and staging of zebrafish embryos followed that described by Kimmel et al. [4]. To obtain eggs, zebrafish at 3 to 6 months of age were randomly selected from a breeding stock and placed in a shallow water breeding tank the night before embryo collection (2 males and 2 females). A divider was used to separate the males and females overnight and were removed at 8:00 am when the facility lights turned on and spawning occurred. Typically, two breeding tanks are prepared the night before to maximize eggs and breeders are bred once a week. Eggs were rinsed with ERM several times and methylene blue (0.00001%) was added as antifungal then eggs sorted under a light microscope in the mornings. All experiments were conducted at pH value of 7.5 ± 0.5, a conductivity value of 600 ± 100 μS /cm, light/dark cycle of 14:10 h, dissolved oxygen concentration ~80% air saturation, and temperature of 27 ± 1 °C. Institutional Animal Care and Use Committee of University of Florida approved all experiments (UF IACUC#201708562).

2.3 Metyltetraprole exposure regime

Fertilized and normally developing eggs were selected at ~6 h post-fertilization (hpf) using a dissecting microscope. Zebrafish eggs were assigned in random fashion into experimental groups (ERM, 0.1% DMSO, or one dose of 0.1, 1, 10, or 100 μg/L MTP). Six experiments were conducted using embryos that were generated from separate breeders of fish. For each experiment, there were 4 replicate breakers for each experimental group containing 20-40 embryos and 20 mL of ERM. Following the addition of chemicals to the water, the glass beakers were placed into an incubator that was maintained at 27 ± 1 °C.

Academic Editor: Firstname Lastname

Received: date
Revised: date
Accepted: date
Published: date

Citation: To be added by editorial staff during production.

Copyright: © 2025 by the authors. Submitted for possible open access publication under the terms and conditions of the Creative Commons Attribution (CC BY) license (https://creativecommons.org/licenses/by/4.0/).

Each day, data related to mortality, deformities, hatch, as well as images using an EVOS™ FL Auto Imaging System (ThermoFisher Scientific, USA) were collected. Deformity assessments included the presence of spinal lordosis and edema (yolk sack/pericardial), as well as hyper/hypopigmentation over the duration of the exposure. Exposure solutions were prepared fresh daily from a stock stored at -20°C in glass amber vials and water was renewed every day at 90% water change.

2.4 Reactive oxygen species

Embryos were obtained for ROS assessment as outlined above in section 2.2. Embryos at 6 hours post-fertilization were washed 3 times in ERM containing 0.0001% (w/v) methylene blue and fertilized eggs were distributed evenly among small petri dishes using sterile micropipettes, each containing ~30 embryos in 20 mL of the assigned chemical concentration. The exposure concentrations were 0.1% DMSO, 1 μg/L, 10 μg/L, or 100 μg/L MTP (n=4 petri dishes per experimental group). Following exposure, zebrafish larvae were transferred to 1.7 mL microcentrifuge tubes, flash frozen in liquid nitrogen, and homogenized in 200 μL of ice-cold PBS. Samples were then centrifuged at 12,000 g for 20 min at 4° C (LYNX6000, Thermo Scientific). For the ROS fluorescence assay, 20 μL of supernatant was transferred to a black fluorescence plate and incubated at room temperature for 5 minutes. After incubation, 8.3 μL of H2-DCFDA (1 mg/mL) and 200 μL of PBS were added. The mixture was incubated in the dark for 30 minutes at 37° C ±1.0° C, and then the contents were measured with an excitation at 485 nm and emission at 520 nm using a multi-detection microplate reader (New Synergy™ 4, BioTek). Total protein was determined for each sample using a BCA assay (Thermo Scientific, ROS levels were expressed as normalized signal intensity/(μg/mL) protein.

2.5 Real-time PCR

Zebrafish embryos at 6 hpf were exposed to 0.1% DMSO, ERM, or one dose of 0.1, 1, or 10 μg/L MTP for gene expression analysis. Each beaker contained 20 embryos and exposure conditions were maintained as that above. Following the 7-days exposure period, larvae were pooled within a beaker, subjected to liquid nitrogen, and placed at −80 °C for RNA extraction. Extraction of RNA from larvae pools was performed using 500 mL TRIzol® Reagent (Life Technologies, Carlsbad, CA, USA) as per manufacturer's protocol. Samples were DNAse treated with DNA TURBO (Ambion). Qubit BR RNA kit was used as per manufacturer's instruction with Qubit® 3.0 Fluorometer (Q33216, Invitrogen) to determine the approximate quantity of RNA in each sample. The concentrations ranged approximately 50-200 ng/ul sample. The cDNA synthesis was performed using ~750 ng of column purified RNA using iScript (BioRad) following the manufacturer's protocol in a final sample volume of 15 μL. Once prepared, samples were placed into a T100™ Thermal Cycler (BioRad, USA). The cDNA was generated using the following steps: 25 °C for 5 min, 42 °C for 30 min, 85 °C for 5 min, and 4 °C for 5 min. Prior to real-time PCR, cDNA stocks were diluted 1:25 in RNAse-DNase free water. The no reverse transcriptase (NRT) controls were prepared in the same way as above without enzyme using 4 randomly selected RNA samples.

Real-time PCR was performed using the CFX Connect™ Real-Time PCR Detection System (BioRad) with SsoFast™ EvaGreen® Supermix (BioRad, Hercules, CA, USA), 200-300 nM of each forward and reverse primer, and 3.33 μL of diluted cDNA. The two-step thermal cycling parameters were as follows: initial 1-cycle Taq polymerase activation at 95 °C for 30 s, followed by 95 °C for 5 s, and 60 °C primer annealing temperature for 5 s. After 40 cycles, a dissociation curve was generated, starting at 65.0 and ending at 95.0°C, with increments of 0.5 °C every 5 s. Primers used in this study were obtained

from published literature (Supplemental Table S1). Three housekeeping genes (ribosomal subunit 15, *rps15,* 18s ribosomal rRNA, *18s,* and beta actin, *b*-actin) were used to normalize expression levels of all target genes. Target genes included catalase (*cat*), cytochrome c oxidase subunit 5Aa (*cox5a1),* cytochrome C oxidase subunit 4I (*cox4i1), ,* cytochrome c1 (*cyc1*), mitochondrially encoded NADH:Ubiquinone oxidoreductase core subunit 1 (*mt-nd1),* mitochondrially encoded NADH:Ubiquinone oxidoreductase core subunit 2 (*mt-nd2),* mitochondrially encoded NADH:Ubiquinone oxidoreductase core subunit 3 (*mt-nd3),* ubiquinone oxidoreductase core subunit S3 (*ndufs3*), superoxide dismutase 1 (*sod1) (Cu/Zn SOD), s*uperoxide dismutase 2 (*sod2) (Mn SOD),* ubiquinol-cytochrome c reductase core protein 2b (*uqcrc2b*)*,* ubiquinol-cytochrome c reductase binding protein (*uqcrb*), ubiquinol-cytochrome c reductase core protein 2 (*uqcrc2*), and ubiquinol-cytochrome c reductase hinge protein *(uqcrh*). Normalized expression was obtained for each target gene using CFX Manager™ software (v3.1) (baseline subtracted) and the Cq method was employed. The qPCR analysis included four NRT samples and two NTC samples. Negative controls indicated that RNA column purification and DNase treatment sufficiently removed gDNA. Sample sizes ranged between 3 to 7 across treatments for gene expression analysis. All primers used in the qPCR analysis amplified one product, indicated by a single melt curve.

2.6 Locomotor activity

Experiments were performed to test the dark photokinesis response in larvae following exposure. Three trials were conducted. Fish were exposed continuously for 7 days with 90% daily water changes as described above at temperature of 27 ± 1 °C and photoperiod of 14:10 h. In each trial, zebrafish embryos at 6 hpf were randomly assigned to an experimental group of ERM, 0.1% DMSO, or one dose of 0.1, 1, or 10 μg/L MTP. Each beaker contained 10 zebrafish embryos and 10 mL of ERM. In mid-afternoon, 4 normally developed larvae were selected from each replicate beaker and placed into a 96-well plate (n=20-24 individuals/treatment). Each well contained 200 μL of ERM. The 96-well plate was placed into DanioVision™ Observation Chamber (Noldus Information Technology, Leesburg, VA) with an infrared analog camera (25 frames/ second) to track the activities of zebrafish larvae. The assay proceeded as per our previous methods [5].

2.7 Statistical analysis

Statistical analysis and graphing were conducted with GraphPad PRISM V10 (La Jolla, CA, USA). A Mantel-Cox test was employed to analyze survival. For ROS (log transformed), gene expression analysis (log10 transformed data), and locomotor activity, a One-Way ANOVA was employed followed by a Dunnett's post hoc test. For behavior, each time period (3 dark and 2 light periods) were analyzed as a discrete response. The DMSO solvent group was considered the control for comparison. There was no difference between the negative control (ERM only) and DMSO control for any endpoint. For all endpoints, the significance of difference was considered at $p < 0.05$.

1. Westerfield, M. *A Guide for the Laboratory Use of Zebrafish (Danio rerio)*; University of Oregon Press: Eugene, OR, USA, 2000.
2. Cao, F.; Souders, C.L., II; Li, P.; Pang, S.; Liang, X.; Qiu, L.; Martyniuk, C.J. Developmental neurotoxicity of maneb: Notochord defects, mitochondrial dysfunction and hypoactivity in zebrafish (*Danio rerio*) embryos and larvae. *Ecotoxicol. Environ. Saf.* **2019**, *170*, 227–237.
3. Perez-Rodriguez, V.; Souders, C.L., II; Tischuk, C.; Martyniuk, C.J. Tebuconazole reduces basal oxidative respiration and promotes anxiolytic responses and hypoactivity in early-staged zebrafish (*Danio rerio*). *Comp. Biochem. Physiol. Part. C Toxicol. Pharmacol.* **2019**, *217*, 87–97.

4. Kimmel, C.B.; Ballard, W.W.; Kimmel, S.R.; Ullmann, B.; Schilling, T.F. Stages of embryonic development of the zebrafish. *Dev. Dyn.* **1995**, *203*, 253–310.
5. Liang, X.; Adamovsky, O.; Souders, C.L., II; Martyniuk, C.J. Biological effects of the benzotriazole ultraviolet stabilizers UV-234 and UV-320 in early-staged zebrafish (*Danio rerio*). *Environ. Pollut.* **2019**, *245*, 272–281.
