# Supplementary material for: Toxicity Assessment of Metyltetraprole, a Novel Fungicide Inhibitor, to Embryo/Larval Zebrafish (Danio rerio)
Source: Toxics. 2025 Jul 28;13(8):634. doi: 10.3390/toxics13080634 (PMC12390205; doi:10.3390/toxics13080634)
Supplement: Supplementary file 1 [file toxics-13-00634-s001.zip › Supplemental Tables.pdf]

Supplemental Tables:

Supplemental Table S1. Primers used for real-time PCR analysis.

Gene name	Gene Symbol	Forward (5' to 3')	Reverse (3' to 5')	Reference
beta-actin	*bactin*	CGAGCAGGAGATGGGAACC	CAACGGAAACGCTCATTGC	Wang et al., 2018 [1]
catalase	*cat*	CTCCTGATGTGGCCCGATAC	TCAGATGCCCGGCCATATTC	Sarkar et al., 2014 [2]
cytochrome c oxidase subunit 4I1	*cox4ia*	GGCAAGTACGGCATTTCGTC	CGACCTTCGCAACTCCATGT	NC_044190.1
cytochrome c oxidase subunit 5A	*cox5a1*	AAGCATAGATGTCTACGATTGTGTGAG	AGGCCAATTAAATAGAACACAAACAC	Duggan et al., 2011 [3]
cytochrome c-1	*cycl1*	ACTTAGCCAACCAGGAGCAC	GGGTGGAAGAAGTCAGAAGC	NC_007117.7
NADH dehydrogenase 1, mitochondrial	*mt-nd1*	AGCCATCTCAAGCCTAGCAG	ATTGTTTGCGCTACAGCTCG	NC_002333.2
NADH dehydrogenase 2, mitochondrial	*mt-nd2*	GACCTACCAGCCACAGCTAC	TTGGGTCGTTTGTACCCGTC	NC_002333.2
NADH dehydrogenase 3, mitochondrial	*mt-nd3*	ACCACTCCCATGAGGAGATCA	CTTGGGCTCATTCGTAGGCT	NC_002333.2
ubiquinone oxidoreductase core subunit S3	*ndufs3*	CATCCCTTCAAAGCAGAACC	AGGCACAGAGGAGTCAAGAG	NC_007118.7
ribosomal protein S18 (rps15)	*rps15*	CTCAGGAGGAAGCAGCAGTCTCT	CCATGTCTCTCAGGTGAGTTTTGAC	(Marín-Juez et al., 2015) [4]
ribosomal protein S18 (rps18)	*rps18*	TCGCTAGTTGGCATCGTTTATG	CGGAGGTTCGAAGACGATCA	McCurley and Callard, 2008 [5]
superoxide dismutase 1	*sod1 (Cu/Zn SOD)*	CAACACAAACGGCTGCATCA	TTTGCAACACCACTGGCATC	Sarkar et al., 2014 [2]
superoxide dismutase 2	*sod2 (Mn SOD)*	AGCGTGACTTTGGCTCATTT	ATGAGACCTGTGGTCCCTTG	Sarkar et al., 2014 [2]
ubiquinol-cytochrome c reductase core protein 2	*uqcrc2*	GACCTCACGGGAAGGGTGAA	TCAGTGTGCTGGTGCTGCTG	NM_001001589
ubiquinol-cytochrome c reductase core protein 2b	*uqcrc2b*	CTGTCAACGATGTAATCAA	CCTCAGAACTCTCAATAGA	NC_007123.7

ubiquinol-cytochrome c reductase binding protein	*uqcrb*	GGGCAGTGATGTGAAAGAGG	AAGGATCTGGTGCTTCATGG	NC_007130.7
ubiquinol-cytochrome c reductase hinge protein	*uqcrh*	ATGATCACGAACGGAGAACC	CCGTCTCCTCACACTTCTGC	NC_007117.7

1. Wang, X.H.; Souders, C.L., II; Zhao, Y.H.; Martyniuk, C.J. Paraquat affects mitochondrial bioenergetics, dopamine system expression, and locomotor activity in zebrafish (*Danio rerio*). *Chemosphere* **2018**, *191*, 106–117.
2. Sarkar, S.; Mukherjee, S.; Chattopadhyay, A.; Bhattacharya, S. Low dose of arsenic trioxide triggers oxidative stress in zebrafish brain: Expression of antioxidant genes. *Ecotoxicol. Environ. Saf.* **2014**, *107*, 1–8.
3. Duggan, A.T.; Kocha, K.M.; Monk, C.T.; Bremer, K.; Moyes, C.D. Coordination of cytochrome c oxidase gene expression in the remodelling of skeletal muscle. *J. Exp. Biol.* **2011**, *214*, 1880–1887.
4. Marín-Juez, R.; Rovira, M.; Crespo, D.; Van Der Vaart, M.; Spaink, H.P.; Planas, J.V. GLUT2-mediated glucose uptake and availability are required for embryonic brain development in zebrafish. *J. Cereb. Blood Flow. Metab.* **2015**, *35*, 74–85.
5. McCurley, A.T.; Callard, G.V. Characterization of housekeeping genes in zebrafish: Male-female differences and effects of tissue type, developmental stage and chemical treatment. *BMC Mol. Biol.* **2008**, *9*, 102.
